# Data Lake, Data Warehouse, Datamart, and Feature Store: Their Contributions to the Complete Data Reuse Pipeline

**DOI:** 10.2196/54590

**Published:** 2024-07-17

**Authors:** Antoine Lamer, Chloé Saint-Dizier, Nicolas Paris, Emmanuel Chazard

**Affiliations:** 1 Univ. Lille, CHU Lille, ULR 2694-METRICS, Centre d'Etudes et de Recherche en Informatique Médicale, Lille, France; 2 Fédération régionale de recherche en psychiatrie et santé mentale des Hauts-de-France, Saint-André-lez-Lille, France; 3 InterHop, Rennes, France

**Keywords:** data reuse, data lake, data warehouse, feature extraction, datamart, feature store

## Abstract

The growing adoption and use of health information technology has generated a wealth of clinical data in electronic format, offering opportunities for data reuse beyond direct patient care. However, as data are distributed across multiple software, it becomes challenging to cross-reference information between sources due to differences in formats, vocabularies, and technologies and the absence of common identifiers among software. To address these challenges, hospitals have adopted data warehouses to consolidate and standardize these data for research. Additionally, as a complement or alternative, data lakes store both source data and metadata in a detailed and unprocessed format, empowering exploration, manipulation, and adaptation of the data to meet specific analytical needs. Subsequently, datamarts are used to further refine data into usable information tailored to specific research questions. However, for efficient analysis, a feature store is essential to pivot and denormalize the data, simplifying queries. In conclusion, while data warehouses are crucial, data lakes, datamarts, and feature stores play essential and complementary roles in facilitating data reuse for research and analysis in health care.

## Introduction

**Figure 1.**
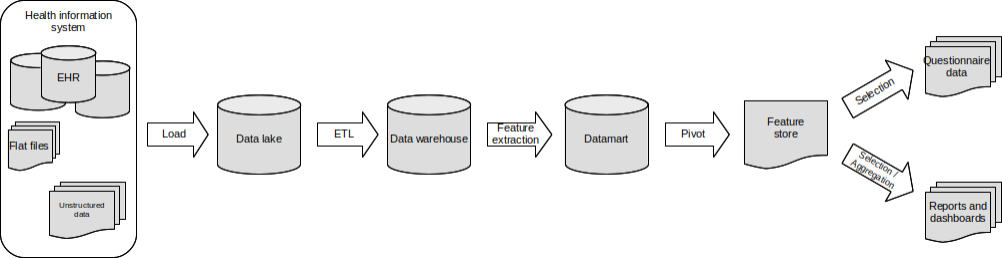
Components of the complete pipeline for data reuse. EHR: electronic health records; ETL: extract-transform-load.

Over the last few decades, the widespread adoption and use of health information systems (HISs) have transitioned a substantial amount of clinical data from manual to electronic format [1]. HISs collect and deliver data for care, administrative, or billing purposes. In addition to these initial uses, HISs also offer opportunities for data reuse, defined as “non-direct care use of personal health information” [2], such as research, quality of care, activity management, or public health [3]. Hospitals have gradually adopted data warehouses to facilitate data reuse [4,5]. Even if the data warehouse is a popular concept, data reuse is not limited to feeding and querying a data warehouse. In this viewpoint, our objective is to outline the different components of the data reuse pipeline and how they complement and interconnect with each other. This definition is derived from our personal experiences and insights gained through collaboration with colleagues at various institutions [5-8]. Additionally, we draw on the collective experiences shared by professionals in the field, contributing to a comprehensive understanding of data reuse practices in diverse health care settings. The pipeline is illustrated in Figure 1 and detailed below. Table 1 compares characteristics of each component. Last, Multimedia Appendix 1 provides examples of data, structures, and architectures for each component of the data reuse pipeline.

**Table 1.** Characteristics of each component of the data reuse pipeline.

Characteristics	Software	Data lake	Data warehouse	Datamarts	Feature store
Content	Data and metadata	Data and metadata	Data	Features	Features and metadata about feature
Architecture	Distributed	Centralized	Centralized	Centralized	Centralized
Detail level	Fine-grained	Fine-grained	Fine-grained	Aggregated	Aggregated
Data	Raw	Raw	Cleaned	Cleaned	Cleaned
Nomenclature	Heterogeneous	Heterogeneous	Standardized	Standardized	Standardized
Data model	Normalized	Normalized	Normalized	Normalized	Denormalized
Data structure	Row-oriented	Row-oriented	Row-oriented	Row-oriented	Column-oriented
Purpose	Transactional software purpose	Ad hoc exploratory queries	All purposes	Prespecified purpose	Prespecified purpose

## Ethical Considerations

This study does not include human participants research (no human participants experimentation or intervention was conducted) and so does not require institutional review board approval.

## Health Information System

The raw data stored in the HIS are distributed across multiple software, making it impossible to cross-reference information between sources due to variations in data formats, ranging from tabular to hierarchical structures and free text [9]. Different technologies and distinct identification schemes for patients, admissions, or any other records compound the complexity. Additionally, direct write access to the software databases is typically unavailable, as software editors rarely grant such privileges to prevent any potential disruption to routine software operation. In transactional software databases, data consist of meticulously organized and highly accurate records presented in rows. These records are collected with great precision to fulfill the specific functions of the software. Alongside the data, a wealth of metadata is also present, including information regarding data collection (eg, information on the individuals inputting data, record timestamps, and biomedical equipment identifiers), as well as software configurations. Notably, a significant portion of these metadata may not be directly relevant to our research purposes, as they primarily support the routine functioning of the software.

## The Data Lake

An optional first component of a comprehensive data reuse pipeline is the data lake [10-14]. A data lake is a centralized, flexible, and scalable data storage system that ingests and stores raw data from multiple heterogeneous sources in its original format [12,15]. Data are stored in a fine-grained, row-oriented, and raw format, in a secure and cost-effective environment. These raw data still encompass diverse formats, from structured data to unstructured text documents, images, songs, videos, and sensor data, ensuring that a wide spectrum of information is readily available for various data analytics endeavors [12].

The technologies implemented for the data lake can include the usual relational databases, such as PostgreSQL or Oracle, but also NoSQL databases and big data technologies, such as the Hadoop Distributed File System or Apache Hudi for the storage and Apache Spark, Hadoop MapReduce, or Apache Kudu for the data processing.

Unlike structured data typically integrated into data warehouses, the data lake refrains from immediate structuring or transformation, allowing for a more agile and adaptable approach. This flexibility enables exploration, manipulation, and, if necessary, transformation of the data to fulfill specific research or analytical requirements. By delaying the application of predefined data models, the data lake cultivates an environment where information can be uncovered without predetermined hypotheses. This includes insights that may not have been evident during the initial phases of data collection and storage. The system further facilitates on-the-fly query processing and data analysis [12,15].

In a data pipeline without a data lake, it is essential to finalize the extract-transform-load (ETL) process before leveraging the data. This introduces a time delay, as it necessitates identifying relevant data in the HIS, updating the data warehouse data model for their accommodation, and subsequently designing and implementing the ETL.

In addition, when interpreting the results, if it becomes apparent that relevant data are missing for the analysis, it requires updating both the ETL process and the data model to incorporate the missing data. This iterative cycle of identifying, modifying, and reimplementing can lead to prolonged timelines and may hinder the agility of the data analysis process. Therefore, a data lake approach proves advantageous in providing a more flexible and dynamic environment for data exploration and analysis, potentially avoiding some of these challenges encountered in a traditional pipeline.

## The Data Warehouse

The data warehouse stands as the most prevalent component of the pipeline and acts as a centralized repository of integrated data from 1 or more disparate sources [5,8,16-19]. It stores historical and current fine-grained data in a format optimized for further use. This involves a single storage technology, a consistent naming convention for tables and fields, and coherent identifiers across data sources. This is a departure from the data lake where all these elements varied between sources.

The data warehouse is supplied through an ETL process [9,18]. The primary objective of this process is to select and extract relevant data from the HIS or other external resources [19]. During this initial phase, the majority of metadata linked to software operations (such as usage logs or interface settings), monitors, and individuals inputting data are usually excluded. Indeed, these types of metadata do not relate to patient care information and would introduce an unnecessary volume of data. Subsequently, the ETL process enhances the raw data by identifying and correcting any abnormal or erroneous information. Following this refinement, data are integrated into a unified data model independent of the source software [9,19]. Notably, there is a strong focus on harmonizing identifiers from diverse data sources to ensure data integrity and streamline queries involving information from multiple origins. The ETL process is also responsible for regularly updating the data warehouse with new information recorded in the original data sources.

The data warehouse, as a relational database, is typically implemented using systems like PostgreSQL, Oracle, SQL Server, Apache Impala, or Netezza. However, for a data warehouse, exploring NoSQL technologies such as MongoDB, Cassandra, or Couchbase can also be interesting, offering advantages in handling unstructured or semistructured data, and providing scalability for large-scale data storage and retrieval [20]. The ETL process can be developed using 2 types of technologies. The first one, with programming languages such as R (R Core Team), Python (Python Software Foundation), or Java (Oracle Corporation), can be used, coupled with a scheduler like Apache Airflow (Apache Software Foundation), to organize the execution of scripts and retrieval of logs and error messages. The second kind of application is graphical user interface software, such as Talend (Talend) or Pentaho (Hitachi Vantara). They do not require programming capacities, because graphical components, corresponding to data management operations, are organized through a drag-and-drop interface.

To foster collaboration among institutions and facilitate the sharing of tools, methods, and results, several initiatives have emerged to offer common data models (CDM). As a result, table and field names are standardized following a common nomenclature, and local vocabularies and terminologies are mapped to a shared vocabulary. Among these CDMs, the Observational Medical Outcomes Partnership CDM was developed by the Observational Health Data Sciences and Informatics community, which brings together multiple countries and thousands of users [21] and led to methodological and practical advancements [22,23].

As a result, the data warehouse functions as a unified, centralized, and normalized repository, for both fined-grained historical data and metadata, and continues to present information in a row-oriented format. The modeling approach presented by Inmon [24] and described as a “subject-oriented, nonvolatile, integrated, time-variant collection of data” implies that data are stored persistently without any assumptions as to their future use, thus remaining open-ended in their usage.

## The Datamarts

While the data warehouse serves as a unique standardized repository, primarily dedicated to data storage, querying these data can be time-consuming due to the volume and distribution of data in the relational model. Furthermore, raw data integrated into the data warehouse may not be readily aligned with specific research or analytical questions, as these data lack the necessary aggregated features. For instance, the data warehouse retains all biological measurements (eg, potassium and sodium), while what will be stored in the datamart are the features related to the biology values, such as the occurrence of hypokalemia, hyperkalemia, hyponatremia, or hypernatremia. Thus, the datamart acts as a dedicated resource for transforming the data into usable and meaningful information [19,25,26]. This transformation process involves feature extraction, achieved through the application of algorithms and domain-specific rules [6,7]. The outcome is data that are tailored to address specific research questions or analytical needs. For instance, within a clinical setting, the datamart can convert raw mean arterial pressure values into a format suitable for detecting perioperative hypotension [5].

Moreover, datamarts can be organized in the form of online analytical processing (OLAP) cubes, offering a multidimensional view of the data [27]. This cubical structure allows for in-depth analysis, enabling users to efficiently explore and navigate across various dimensions such as time, geography, or specific categories, gaining profound and contextualized insights. These datamarts are often modeled in either a snowflake or star schema, optimizing their structure for the creation of OLAP cubes. The star schema, with its central fact table surrounded by dimension tables, or the snowflake schema, which further normalizes dimension tables to minimize data redundancy, both serve to facilitate the creation of these OLAP cubes. Such schemas play a pivotal role in enhancing the efficiency of multidimensional data analysis within the OLAP environment, providing a structured framework for faster and more comprehensive insights.

In the context of health care, an example of an OLAP cube could encompass dimensions such as patient (eg, age and gender); time (eg, admission and discharge dates); medical conditions (eg, primary and secondary diagnoses and medical procedures); hospital unit (eg, information on services, departments, and bed types); health care provider (eg, physicians); and outcome (eg, length of stay, treatment outcomes, and medical costs). The cube would include various facts, such as the number of patients, average length of stay, and average treatment costs. This multidimensional structure allows health care professionals to conduct in-depth analyses, explore trends over time, compare costs across different hospital units, and assess the impact of medical interventions on patient outcomes [19,28].

Datamarts, owing to the structured nature of their data, are typically stored on relational databases (eg, PostgreSQL, Oracle, and SQL Server) [25]. In the case of OLAP cubes, this may include Apache Kylin or other proprietary OLAP tools built on relational databases [28,29].

In contrast to Inmon’s [24] approach, the Kimball [9] bottom-up approach places datamarts at the core, with their design driven primarily by business requirements. However, by directly developing datamarts, the Kimball approach may overlook some crucial data that were not initially identified as relevant during the business requirements phase.

As a result, the datamart stands as a centralized component for cleaned and aggregated features for dedicated purposes, still stored in row-oriented structure.

## The Feature Store

The feature store addresses the limitations of the traditional row-oriented, relational database structure typically used in datamarts. This architecture, which relies on multiple tables, may not fully meet various analytical requirements. For instance, effective statistical analysis often necessitates a single, flat file with column-oriented variables, mandating the transformation of data from a row-based to a column-based format within the feature store. This process streamlines data access, simplifying complex queries into straightforward selections from a single table. Consequently, the feature store emerges as a centralized repository housing well-documented, curated, and access-controlled features. In addition to features extracted from datamarts, which are often calculated by algorithms derived from business rules, the feature store can also receive features generated by machine learning algorithms [30].

The design of the feature store aims to provide data scientists with direct access to these features, eliminating the need for additional data cleaning, aggregation, or pivoting [31]. This specialized role enhances efficiency and promotes the use of high-quality, analysis-ready data, significantly contributing to the effectiveness of data-driven research in the health care organization. Notably, the feature store not only stores the features themselves but also their associated metadata, documenting how they were calculated and used [31]. It ensures the preservation of all feature versions, guaranteeing the reproducibility of analyses.

When derived from business rules, features are stored in relational databases (eg, PostgreSQL, Oracle, and SQL Server) or in a NoSQL data store such as MongoDB to also store metadata. When features originate from machine learning models, they are stored and shared from big data platforms such as Databricks or Hopsworks [30,32].

As the final component of the data reuse pipeline, the feature store plays a pivotal role in various analytical applications within the health care organization. It significantly contributes to the creation of insightful dashboards and automated reports, delivering real-time and historical information. In research, its most crucial contribution lies in generating denormalized flat tables, similar to questionnaire data tailored for statistical analyses.

## Conclusions

In this opinion paper, we propose standardized nomenclature and definitions for the components of a data reuse pipeline. Table 2 summarizes the advantages and limitations of each component in this pipeline.

While the data warehouse serves as a necessary initial stage, the integration of datamarts and a feature store enhances its effectiveness. Datamarts compute pertinent information from raw data, while the feature store organizes it into columns, streamlining data set construction. Additionally, the data lake emerges as a valuable resource for storing raw data in a single location, allowing for exploitation without having to wait for the entire pipeline to be developed.

Notably, in a data pipeline without a data lake, the requirement to complete the ETL process before analysis introduces delays. This involves identifying relevant data in the HIS, adapting the data warehouse data model, and implementing the ETL. Additionally, discovering missing data during result interpretation prompts iterative updates to both the ETL process and the data model, potentially prolonging timelines and hindering data analysis agility.

It is important to emphasize that the specific components and their characteristics described here are not rigidly fixed and can vary based on the unique organizational needs and configurations. For instance, the inclusion of a data lake and feature store is often discretionary, influenced by factors such as the scale and intricacy of source data, the quantity of features, the scope of research projects, the team’s size, and the imperative for study reproducibility over time.

**Table 2.** Advantages and disadvantages of the components of the data reuse pipeline.

Component	Advantages	Disadvantages
Data lake	All data sources on the same server Independence from source software On-the-fly query processing and data analysis without the need for the complete development of an extract-transform-load (ETL) process	Inconsistencies in data formats and structures Lack of standard schema can make querying complex Analyses reproducibility
Data warehouse	Querying data from both administrative and biology systems is facilitated by the unified data model (ie, data from both systems are linked, and the model conventions are consistent) Relevant data are retained at the finest level of detail (eg, dates, diagnoses, and all biology values), enabling the answering of numerous questions without necessarily identifying them beforehand	ETL process must be implemented to standardize the data Multidimensional data model with several statistical units Fine-grained data is not directly usable and adapted for statistical analysis or decision-making
Datamarts	Features are ready to be used directly	Features are still organized with a row-format (ie, 1 feature per row) in several datamarts
Feature store	Using features directly, without the need for data management tasks such as joining datamarts or pivoting to reorganize features into columns	Having developed the entire pipeline beforehand

## Supplementary material

10.2196/54590 Multimedia Appendix 1 Comparison of data, structures, and architectures of components of the data reuse pipeline.

## Abbreviations

CDM: common data model
ETL: extract-transform-load
HIS: health information system
OLAP: online analytical processing
