# Supplementary material for: Data Lake, Data Warehouse, Datamart, and Feature Store: Their Contributions to the Complete Data Reuse Pipeline
Source: JMIR Med Inform. 2024 Jul 17;12:e54590. doi: 10.2196/54590 (PMC11267403; doi:10.2196/54590)
Supplement: Multimedia Appendix 1 [file medinform-v12-e54590-s001.docx]

# Hospital Information Management System

In the Hospital Information System (HIS), the hospital administration system contains data related to the patient (sex and birth_date), the hospital stay (admission date and discharge date), the diagnoses and other types of data not represented in this exemple. The biology system comprises biological results, accompanied by details such as the measured parameter, the professional who performed the measurement, the analysis medium, and the automated system used.

The two systems are hosted on separate servers. The table and field naming conventions are heterogeneous, and the database technologies may be different, depending on the software editor. For instance, the hospital stay identifier is labeled as "stay_id" in the administration system and "hospital_stay_id" in the biology system, and the hospital administration system is hosted in a PostgreSQL database whereas the biology system is hosted on a SQL Server database. It's worth noting that hospital stay identifiers have distinct values across different software systems, complicating direct linkage. Finally, routinely querying either database with complex requests could slow down the software's performance.

# Data lake

In the data lake, data is host on the same server. In this way, data can be queried without the risk of causing slowdowns during routine software use. However, data are still organised according to their origin data model and the nomenclature and technologies remain heterogeneous.

Example query: Identify stays during which potassium levels were measured.

# Data warehouse

In the data warehouse, data from the two software sources are now integrated within a unified data model, following a consistent naming convention. Consequently, data from the biology system is associated with patient information from the administrative system, ensuring uniformity and alignment of hospital identifier labels and values. Dates are standardized to a consistent format. Only relevant tables and variables for reuse are retained in the data warehouse. Metadata tables, such as the list of biological measurement devices or operators, are not kept.

Example query: Extract the biology results of hospital stays in 2020 and associate them with the documented diagnoses during those stays.

# Datamart

The features related to the patient and the stay have been calculated and extracted from the initial tables, such as age at admission, year of birth, and day of admission. More complex features related to biology have been extracted based on business rules, such as the presence of hypokalemia, hyperkalemia, hyponatremia, hypernatremia, enabling direct responses for analysis needs.

Example queries: Number of patients per day of the week. Number of patients with hypokalemia.

# Feature store

The features are extracted from the datamarts to form a single flat table with all the features required for analyses. The features are now presented as columns, incorporating patient, stay, and biology-related features. The time dependency, which was still present in the datamarts, is reflected in the variable names. For example, hypokalemia_d0 reflects the presence of hypokalemia on day 0 (at admission).

Example query: Compare the age at admission, sex, occurrence of relevant diagnoses, and the occurrence of hypokalemia and hyperkalemia.
